# Pollution Characteristics and Health Risks of Polycyclic Aromatic Compounds (PACs) in Soils of a Coking Plant

**DOI:** 10.3390/toxics12030179

**Published:** 2024-02-27

**Authors:** Yousong Zhou, Yuancheng Li, Donglei Fu, Yongqiang Zhang, Kai Xiao, Ke Jiang, Jinmu Luo, Guofeng Shen, Wenxin Liu, Shu Tao

**Affiliations:** 1 Key Laboratory for Earth Surface and Processes, College of Urban and Environmental Sciences, Peking University, Beijing 100871, China; 1901111768@pku.edu.cn (Y.Z.); 2000013357@stu.pku.edu.cn (Y.Z.); 18817655968@163.com (K.X.); 1800013254@pku.edu.cn (K.J.); gfshen12@pku.edu.cn (G.S.); wxliu@urban.pku.edu.cn (W.L.); taos@pku.edu.cn (S.T.); 2 School of Public Health, Shandong First Medical University (Shandong Academy of Medical Sciences), Jinan 271016, China; liyuancheng@sdfmu.edu.cn; 3 Department of Earth and Atmospheric Sciences, Cornell University, Ithaca, NY 14853, USA; jl3439@cornell.edu

**Keywords:** coking plant, soil parent-PAHs, PAH derivatives, potential sources, health risk

## Abstract

Coke production is an important source of environmental polycyclic aromatic compounds (PACs), including parent polycyclic aromatic hydrocarbons (PAHs) and their derivatives. The focus near coking plants has primarily been on parent-PAH contamination, with less attention given to highly toxic derivatives. In this study, soil samples were collected from both within and outside of a coking plant. The concentrations of parent-PAHs and their derivatives, including methylated-PAHs, oxygenated-PAHs, and nitrated-PAHs, were examined. Spatial interpolation was employed to determine their spatial distribution patterns. Methods for identifying potential sources and conducting incremental lifetime cancer risk analysis were used. This could achieve a comprehensive understanding of the status of PAC pollution and the associated health risks caused by coke production. The concentrations of total PACs inside the plant ranged from 7.4 to 115.8 mg/kg, higher than those outside (in the range of 0.2 to 65.7 mg/kg). The spatial distribution of parent-PAH concentration and their derivatives consistently decreased with increasing distance from the plant. A significant positive correlation (*p* < 0.05) among parent-PAHs and their derivatives was observed, indicating relatively consistent sources. Based on diagnostic ratios, the potential emission sources of soil PACs could be attributed to coal combustion and vehicle emissions, while principal component analysis–multiple linear regression further indicated that primary emissions and secondary formation jointly influenced the PAC content, accounting for 60.4% and 39.6%, respectively. The exposure risk of soil PACs was dominated by 16 priority control PAHs; the non-priority PAHs’ contribution to the exposure risk was only 6.4%.

## 1. Introduction

Polycyclic aromatic compounds (PACs) are ubiquitous hazardous pollutants, frequently measured in the environment, e.g., air, water, soil, sediments, and foods [1]. Natural and anthropogenic sources can produce PACs, with the latter contributing largely to the environment of PACs in certain regions [2]. Major anthropogenic sources are the incomplete burning of carbonaceous fuels in sectors like industry, transportation, and residential combustions. The contamination levels of PACs vary globally by region, and studies from Asia, Europe, and North America indicate higher levels of PACs in urban soils relative to suburban and rural areas [3,4]. Studies indicate that concentrations of PACs either exceed the corresponding environmental standards or reach the high-risk level based on the incremental lifetime cancer risk method [5,6,7,8]. Although carcinogenic PACs, with high toxicity, account for only 5.98% of the total global emissions [9], their metabolites can cause cellular damage, genetic mutations, and cancer after entering the human body [10]. Similarly, derivatives of parent polycyclic aromatic hydrocarbons (PAHs) are commonly present in the environment, with mutagenicity levels up to twice that of the parent-PAHs [11]. Nitrated-PAHs, derivatives of parent PAHs, contribute up to 17% to total cancer risks [12]. Shen et al. [9] have reported that the global emission of 16 PAHs is 520 Gg, with 53.5% coming from Asia and 21.92% coming from China. Such severe PAC pollution may have adverse impacts on human health. It was reported that the lung cancer mortality rate caused by exposure to PACs in the southwestern region of China was 78 per 100,000 individuals from 2005 to 2011 [13]. Epidemiological studies associated with PAHs’ exposure have also been investigated in China. Tian et al. [14] revealed that long-term exposure to PACs among coke oven workers is associated with poor attention and memory performance. A study conducted near petrochemical plants showed that long-term exposure to PACs raises the risk of renal impairment and chronic kidney disease [15]. The health risks associated with PACs are closely related to China’s industrial structure and energy composition.

According to the data from the World Bank in 2020, China ranks as the world’s largest industrial nation, with a manufacturing output value of US$4.18 trillion. Pollution events caused by traditional heavy industry PAC emissions have been continuously investigated since the 1970s. Among those industrial sectors, coking industries are believed to release substantial amounts of PACs, especially in China. It is reported that China’s coke production peaked and reached 438.2 million tons, accounting for 67% of the world’s production in 2018 [16]. A study conducted by Xu et al. [17] estimated that the total amount of PAC emissions in China in 1999 was 9799 tons, with coking emissions accounting for 30.6% of the total. Since 1996, beehive coke ovens began to be banned in China, resulting in a reduction of 17.4 Gg in Benzo(a)pyrene emissions by 2015 [18]. Industrial coking plants above a certain scale have gradually gained attention [19,20]. In the process of urbanization in China, some industrial coking plants have gradually become connected to and even integrated into urban areas, and they share the common characteristics of long service age, high production capacity, and high pollution emissions. Although some of them have undergone upgrades or been completely dismantled, they still pose potential health risks to the surrounding areas [21,22,23,24,25]. A study conducted on a coking plant in southern China [26] shows that although the soil PAC concentration within the factory is significantly higher than outside, the high concentration of PACs (105.9 mg/kg) found outside induces a high-risk level of 2.9 × 10^−4^. Thus, conducting a comprehensive investigation on soil PACs both inside and outside the coking plant is critical for emission source control as well as soil ecological restoration.

Researchers have long been concerned with the status of soil PAC pollution yielded by coking plant emissions, with attention paid to the limited 16 priority control polycyclic aromatic hydrocarbons (referred to as priority PAHs hereafter), as proposed by the United States Environmental Protection Agency (EPA) guidance in the 1970s. However, with the advancement of monitoring techniques, the characteristics of non-priority PAHs have continued to receive attention, mainly due to the high toxicity of certain non-priority PAHs, such as Benzo(e)pyrene, Dibenzo(a,e)fluoranthene, Dibenzo(a,i)pyrene, and 6-nitrochrysene [27,28,29,30]. A study has indicated that when considering only priority PAHs, the respiratory exposure risk achieved over Nanjing City, China, is 5.6 × 10^−5^, but it significantly increases to 4.4 × 10^−4^ when non-priority PAHs are considered [31]. Furthermore, Samburova et al. [32] found that the carcinogenic potential of the 16 particle-bound priority PAHs underestimates 85.6% of the total 88 detected PACs. Therefore, the current characterization studies on PACs based only on the priority PAHs have certain limitations.

In this study, we focus on a combination of “coke production & high population density & the risk of parent-PAHs and their derivatives”. A coking plant in Jinan, China, is targeted. The plant was built in 1970 and is surrounded by residential areas, villages, and arable land. At present, the plant has a total of six coke ovens in operation, and the annual output of coke is 4.12 million tons. In addition to the production of coke, it also produces industrial coke oven gas, coal tar, light benzene, ammonium sulfate, industrial naphthalene, modified asphalt, and 20 other kinds of products. Soil samples were collected both inside and outside the coking plant for three aspects of the work. Firstly, the soil concentrations of parent-PAHs and their derivatives were determined, and their spatial distribution was obtained using spatial interpolation. Secondly, potential sources of soil PACs were identified using diagnostic ratios, while the proportions of primary emissions and the secondary formation of PACs were quantified using principal component analysis–multiple linear regression. Lastly, a risk assessment of PAC exposure was performed using the incremental lifetime cancer risk method, considering parameters such as PAC concentrations, toxicity equivalency factor values, exposure duration, etc. The results were presented in terms of gender, age groups, and exposure pathways. This study aims to provide comprehensive insights regarding the levels, spatial distribution, and human exposure risks of parent-PAHs and their derivatives within the coking plant and its surrounding soils. To the best of our knowledge, research on soil parent-PAHs and their derivatives from coking plants is relatively scarce. Conducting in-depth investigations on these compounds will provide a comprehensive understanding of soil PAC status and yield more reliable results for exposure risk assessment. Results from the study enhance our scientific understanding of the impacts of these specific industry factories on soil quality and human health.

## 2. Materials and Methods

### 2.1. Soil Sampling and Pretreatment

**Figure 1:**
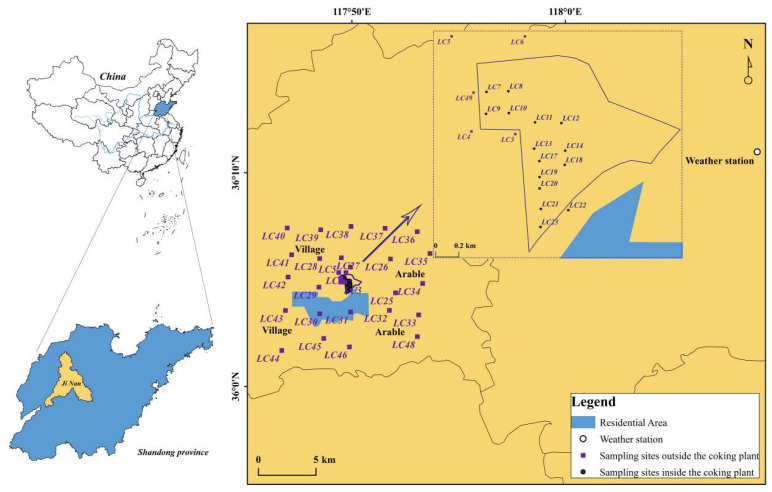
Sampling sites of the study.

In March 2022, we collected 44 surface soil samples (0–10 cm) in the coking plant and its surrounding areas using the grid distribution method, with 15 samples collected from the coking plant (Figure 1). The sampling grid size inside the coking plant is 300 m × 300 m, and the surrounding area is 2 km × 2 km. Before sampling, the overlying vegetation was removed. Five sub-samples were collected from each sampling point within 100 m^2^ and fully mixed. The mixed sample was then placed in an aluminum foil bag and sealed. In the laboratory, samples were freeze-dried for 24 h and were stored at −20 °C until analysis after being ground and sieved (70-mesh).

2.0 g of the soil sample was transferred into a polytetrafluoroethylene microwave tube. 25 mL of the mixed solvent (hexane and acetone, 1:1) and the mixed surrogates (p-terphenyl-d_14_ and 2-fluorobiphenyl for parent-PAHs and methylated-PAHs, and 1-Br-2-nitrobenzene for oxygenated-PAHs and nitrated-PAHs, respectively) were then added for microwave extraction using the CEM Mars Xpress microwave extractor (Matthews, NC, USA, at 1200 W). During the extraction process, the temperature was gradually raised to 100 °C at a rate of 10 °C/min and held at that temperature for 10 min before being cooled for 30 min. The filtered extract was evaporated by a rotary evaporator (N-1100, EYELA, Tokyo, Japan) at 37 °C water bath conditions until 1 mL remained. Then, it was further evaporated after being diluted with 10 mL of hexane until 1 mL remained. Subsequently, purification was performed using a neutral silica gel column (1 cm i.d. × 10 cm length, 6 cm silica gel height) with elution by 50 mL of dichloromethane. The eluent was evaporated to 1 mL and then concentrated to 1 mL after adding 10 mL of hexane. Finally, the mixed internal standards (Naphthalene-d_8_, Acenaphthene-d_10_, Anthracene-d_10_, Chrysene-d_12_, and Perylene-d_12_ for parent-PAHs and methylated-PAHs; 9-nitroAnthracene-d_9_ and 1-nitroPyrene-d_9_ for oxygenated-PAHs and nitrated-PAHs, respectively) were quantitatively added to obtain the solution under test.

### 2.2. PACs Analysis, Quality Assurance (QA), and Quality Control (QC)

In most studies, gas chromatography (GC) or liquid chromatography (LC) coupled with mass spectrometry (MS) was used to analyze PACs. Among them, GC is suitable for volatile and thermally stable compounds, while LC bears the advantages of fast analysis, high resolution, and high sensitivity. GC coupled with MS (GC-MS) is superior to LC due to the characteristics of the GC mobile phase. At present, the combination of capillary GC-MS has become a conventional method to measure PACs. Although there are many interfaces, such as ion beam, thermal spray, electrospray, etc., the choice of the mobile phase of LC-MS is still limited. Compared with MS, tandem mass spectrometry (MS/MS) has higher detection selectivity. To date, many studies have successfully applied GC-MS/MS to measure PACs [33,34,35,36].

In this study, parent-PAHs and methylated-PAHs were analyzed by Agilent (Palo Alto, CA, USA) 6890-5973 GC-MS in electron ionization (EI) mode. One microliter of the eluate was injected in spitless mode, and high-purity helium was used as a carrier gas. The oven temperature was programmed at 70 °C for 1 min, increased to 280 °C at a rate of 5 °C/min, and held for 20 min. Oxygenated-PAHs and nitrated-PAHs were analyzed by 7890-5975 GC-MS in negative ion chemical ionization (NICI) mode. One microliter of eluate was injected in splitless mode, and the carrier gas was also high-purity helium. The oven temperature was programmed at 60 °C for 1 min, increased up to 150 °C at a rate of 15 °C/min, increased to 300 °C at a rate of 5 °C/min, and then held for 15 min.

44 PACs were tested in this study, including parent-PAHs, methylated-PAHs, oxygenated-PAHs, and nitrated-PAHs.

Parent-PAHs: Fluoranthene(FLA), Phenanthrene(PHE), Benzo(b)fluoranthene(BbF), Benzo(a)anthracene(BaA), Naphthalene(NAP), Benzo(a)pyrene(BaP), Benzo(k)fluoranthene(BkF), Chrysene(CHR), Indeno(1,2,3,-cd)pyrene(IcdP), Benzo(ghi)perylene(BghiP), Anthracene(ANT), Fluorene(FLO), Acenaphthene(ACE), Acenaphthylene(ACY), Dibenz(a,h)anthracene(DahA), Pyrene(PYR), Benzo(e)pyrene(BeP), Dibenzothiophene(DIB).

Methylated-PAHs: Retene(RET), 2-Methylnaphthalene(2-MEN), 2-Methylphenanthrene(2-MEP), 2,6-Dimethylnaphthalene(26-DIN), 1,3-Dimethylnaphthalene(13-DIN), 1-Methylphenanthrene(1-MEP), 1-Methylpyrene(1-MPYR), 2-Methylanthracene(2-MEA), 6-Methylchrysene(6-MEC), 3,6-Dimethylphenanthrene(36-DIP), 1-Methylnaphthalene(1-MEN).

Oxygenated-PAHs: anthracene-9,10-dione(OANT), 9-fluorenone(OFLU), benz(a)anthracene-7,12-dione(OBAD), benzanthrone(OBEN).

Nitrated-PAHs: 5-nitroAcenaphthene(5-NACE), 6-nitroBenzo[a]pyrene(6-NBaP), 1-nitroPyrene(1-NPYR), 2-nitronaphthalene(2-NNAP), 1-nitronaphthalene(1-NNAP), 7-nitroBenzo(a)anthracene(7-NBaA), 2-nitroFluorene(2-NFLU), 9-nitroAnthracene(9-NANT), 3-nitroFluoranthene(3-NFLA), 9-nitroPhenanthrene(9-NPHE), 3-nitroPhenanthrene(3-NPHE).

All of the data were subjected to quality control procedures to ensure the correct identification and accurate quantification of the target compounds. All the glassware used in the experiment was ultrasonically cleaned, rinsed with tap water and ultrapure water three times in a turn, and then placed in a muffle furnace at 450 °C for 6 h. Silica gel was burned in a muffle furnace at 450 °C for 6 h before use, and anhydrous sodium sulfate was burned at 650 °C for 10 h. The acetone, dichloromethane, and n-hexane solvents used were all high-performance liquid chromatography (HPLC) grade.

Before the formal determination, the method recovery experiment was tested. At the same time, three unspiked samples and seven spiked samples were analyzed. The recoveries of 44 PACs were in the range of 59% and 133%, which met the accuracy requirements of the actual samples.

Each batch of samples included 10 soil samples and one matrix blank sample. Method detection limits (MDLs) were calculated as the mean matrix blank concentrations added to three times the standard deviation (MDLs = mean matrix blank concentrations + 3SD). However, most of the parent-PAHs and their derivatives were not detected in the matrix blank samples, indicating that the pollution introduced in the analysis process was negligible. In this case, the MLDs were calculated by three times the signal-to-noise ratio. The MLDs of parent-PAHs and methylated-PAHs ranged from 0.1 to 4.4 pg/g, and oxygenated-PAHs and nitrated-PAHs ranged from 1.1 to 333.4 pg/g [37,38]. Other corresponding details about QA/QC can be found in Table S1.

### 2.3. Total Organic Carbon Analysis

The total organic carbon (TOC) fraction in the soil was measured by a TOC analyzer (TOC-V CPH, Shimadzu 5000-A, Shimadzu, Japan). In general, two soil samples of about 50 mg were weighed and placed in two boat-shaped containers. One is determined by total carbon (TC) with WO_3_ as the catalyst, and the reaction temperature was 900 °C; in this reaction, both organic carbon and inorganic carbon were converted to CO_2_, and the TC content (%) was calculated according to the signal in the non-dispersive infrared detector. Dilute phosphoric acid (10%, 0.5 mL) was added to another boat-shaped container, which was run at 200 °C to acidify the soil sample. The inorganic carbon was converted to CO_2_, which was detected in the same detector, and thus, total inorganic carbon (TIC) content was determined. Finally, TOC was obtained by subtracting TIC from TC.

### 2.4. Data Analysis

Exposure risk assessment is estimated by incremental lifetime cancer risk (ILCR). The calculation of ILCR is based on the tested 44 PACs, which are reclassified into priority PAHs and non-priority PAHs in Section 3.3 for discussion. The parameters used are collected from EPA guidance (https://www.epa.gov/risk/regional-screening-levels-rsls-users-guide) (accessed on 16 September 2023) and documents [39,40,41,42]. Before the calculation, the contributions of PACs need to be adjusted by multiplying corresponding Toxic Equivalent Factors (TEFs), which are derived from documents [27,31,32,43,44]. The exposure risk is generally classified into no appreciable risk level, potentially low-risk level, and high-risk level, with corresponding thresholds of <1.0 × 10^−6^, 1.0 × 10^−6^~1.0 × 10^−4^, and >1.0 × 10^−4^. Equations to obtain ILCR by pathways regarding ingestion, dermal contact, and inhalation are as follows:

(1) $$\mathrm{ILCRIng}=\frac{CS\times (CSF_{Ingestion}\times \sqrt[{3}]{\left(BW/70\right)})\times IR_{soil}\times EF\times ED}{BW\times AT\times 10^{6}}$$

(2) $$\mathrm{ILCRDer}=\frac{CS\times (CSF_{Dermal}\times \sqrt[{3}]{\left(BW/70\right)})\times SA\times AF\times ABS\times EF\times ED}{BW\times AT\times 10^{6}}$$

(3) $$\mathrm{ILCRInh}=\frac{CS\times (CSF_{Inhalation}\times \sqrt[{3}]{\left(BW/70\right)})\times IR_{air}\times EF\times ED}{BW\times AT\times PEF}$$

where ILCRIng, ILCRDer, and ILCRInh indicate exposure risk caused by corresponding ingestion, dermal, and inhalation intakes. *CS* is the concentration of individual PAHs (mg/kg); *CSF* = carcinogenic slope factor for corresponding intake pathway (mg/kg·d)^−1^; BW = average body weight (kg); *SA* = dermal surface exposure (cm^2^/d); *AF* = particle-to-skin adherence factor (mg/cm^2^); *ABS* = dermal adsorption fraction; *EF* = exposure frequency (d/year); *ED* = exposure duration (year); *IR* is the intake rate by ingestion (mg/d) or inhalation (m^3^/d); *AT* = average life span (year); *PEF* = soil dust produce factor (m^3^/kg). The values used for ILCR calculation, which were collected from documents [23,41,45,46,47] and EPA (https://www.epa.gov/risk/regional-screening-levels-rsls-users-guide) (accessed on 16 September 2023), are listed in Table 1.

Principal component analysis–multiple linear regression (PCA-MLR) is a commonly used approach for quantitatively analyzing emission sources. In this study, we applied this method to evaluate the impacts of direct emission and secondary formation on soil PACs. PCA is used to reduce original variables to achieve principal components, and then establish the relationship between the principal components (independent variables) and the PAC concentration (dependent variable) [31,48].

y = ∑m_i_*X_i_* + b (4)

where y is the PAC concentration. *X_i_*, with no collinearity, represents PCA factor scores as the independent variables. Equation (4) presents as Equation (5) if the independent and dependent variables are normalized.

z = ∑*B_i_X_i_* (5)

where z is the PAC concentration that is normalized, and *X_i_* indicates the same as that in Equation (4). *B_i_* is the regression coefficient. The relative contribution of source *i*, *Pi*, is a ratio of *Bi* and the sum of *Bi* (Equation (6)).

(6) $$Pi(\%)=\frac{Bi}{\sum Bi}$$

PCA-MLR analysis was conducted by SPSS 24.0, the varimax method was employed for rotation, and eigenvalues exceeding 1.0 were retained.

## 3. Results and Discussion

### 3.1. PAC Concentrations

The surrogate recoveries of 2-fluorobiphenyl and p-terphenyl-d_14_ for parent-PAHs and methylated-PAHs were 77 ± 12% and 79 ± 13%, respectively, and that of 1-Br-2-nitrobenzene for oxygenated-PAHs and nitrated-PAHs ranged from 60% to 105%, all of which were acceptable. In this study, the concentrations of parent-PAHs and their derivatives were obtained by subtracting the matrix blank concentration but not corrected by the recovery rate.

**Figure 2:**
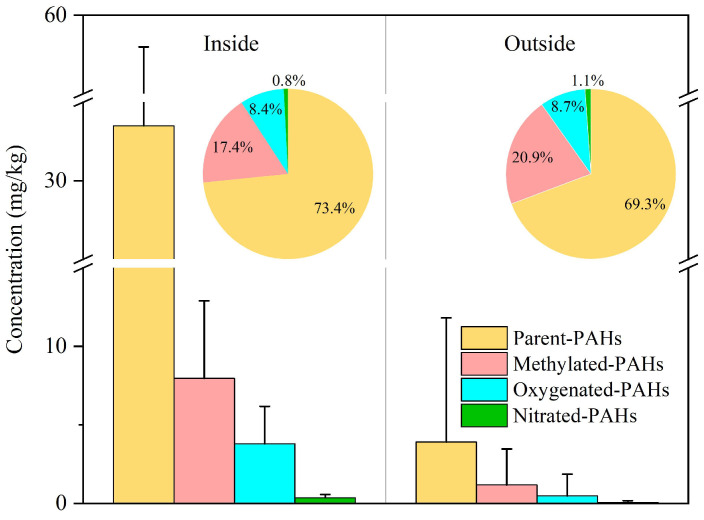
The concentrations of PAC groups and their contributions to total PAC mass concentration inside and outside the coking plant. Error bars represent standard deviations.

Contamination levels of different PAC individuals in soils from inside and outside the coking plant are listed in Table S2. The average concentration of total PACs by sampling sites was 45.6 mg/kg (in the range of 7.4~115.8 mg/kg) in the soil inside the plant but was 5.6 mg/kg (0.2~65.7 mg/kg) in the soil outside the coking plant. Soil PACs, either inside or outside the coking plant, are expected to vary largely in different sites, which will be discussed in Section 3.3. Parent-PAHs still comprised the majority of the total PACs in this study, with the mass fractions at 73.4% and 69.3% in the soils inside and outside the plant, respectively, followed by the methylated-PAHs (Figure 2).

Soil PACs’ contamination levels could be affected by many factors, such as emission intensities, soil properties, local meteorological conditions, service age, and capacity, that influence the dispersion and deposition of atmospheric PACs, thus inducing discrepancies in concentrations as compared to previous studies. Here, the mean soil PAC concentration outside the targeted coking plant (5.6 mg/kg, in the range of 0.2~65.7 mg/kg) is lower than the limitation prescribed by the Risk control standard for soil contamination of development land (GB36600-2018) enacted by the Ministry of Ecology and Environment, China (https://www.mee.gov.cn/ywgz/fgbz/bz/bzwb/trhj/201807/t20180703_446027.shtml) (accessed on 16 September 2023). It is higher than that of the Rong Xin coking plant surroundings located in Shandong province (3.0 mg/kg, in the range of 1.7~4.6 mg/kg, Wu et al. [25]), and coking plant surroundings in Shanxi Province, China (1.0 mg/kg, in the range of 0.2~3.2 mg/kg, Cui et al. [49]). There are numerous studies focused on soil priority PAHs relative to coking plant emissions. Priority PAHs’ concentration in soil can range from 2.5 to 115.0 mg/kg [21,22,25,26,45,50,51,52]. For studies involving PAH derivatives, compared to the available data on oxygenated-PAHs and nitrated-PAHs in soils from urban or rural areas, our results were nearly one–two orders of magnitude higher than those achieved by Cai et al. [53]. In contrast, when compared to the study conducted by Boulange et al. [54], the concentrations of oxygenated-PAHs and nitrated-PAHs obtained here were lower by two orders of magnitude. This notable disparity can be primarily attributed to differences in control facilities, production capacity, and operational lifespan among coking plants.

### 3.2. Composition Profiles of PACs

**Figure 3:**
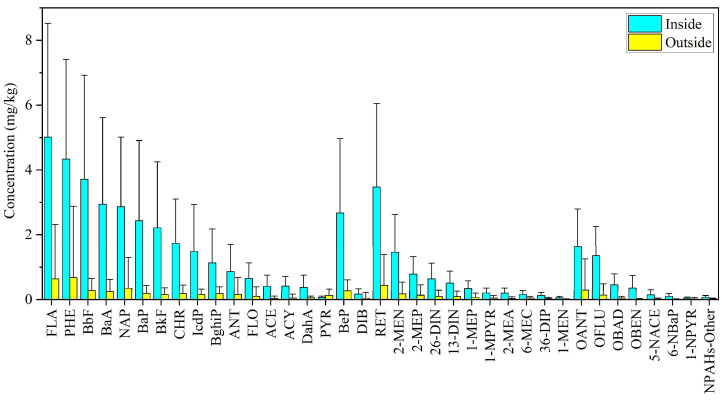
The mass concentration of PAC individuals inside and outside the coking plant. Nitrated-Other indicates the remaining total mass of nitrated derivatives. Error bars represent standard deviations.

Among the parent-PAHs targeted, FLA (11.0%), PHE (9.4%), BbF (8.3%), BaA (6.7%), NAP (5.7%), BaP (5.6%), as well as RET and BeP were more abundant compared to other species. The profile was similar in soils inside and outside the coking plant, indicating the influence of similar sources (Figure 3). Previous studies reported that PHE, FLA, and NAP were also major compounds in coking plants [24,55]. It was noted that RET and BeP were relatively higher compared to other parent-PAHs. These two compounds are not regulated in the priority list, but available studies suggested that they had significant environmental implications in pollution source apportionment and photochemical degradation analysis of PACs in the environment [56,57]. The mean concentration of BeP was 1.1 mg/kg, which is much higher than the BeP levels of 0.01–0.06 mg/kg reported by previous studies in agricultural and urban soils [24,44,53]. Abundant RET was due to prevalent biomass burning in areas nearby [58,59], and it was found to be higher in the soil outside the plant compared to the inside sites.

### 3.3. Spatial Distribution and Influencing Factors

**Figure 4:**
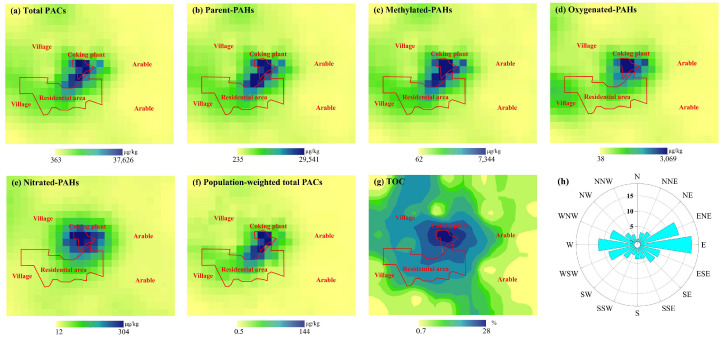
The spatial distribution of total PACs and PAH groups (**a**–**e**), population-weighted total PACs (**f**), and TOC (**g**). The red lines indicate the boundary of the coking plant and the residential area. (**h**) Indicates the wind direction frequency (%) from 2018 to 2022.

**Figure 5:**
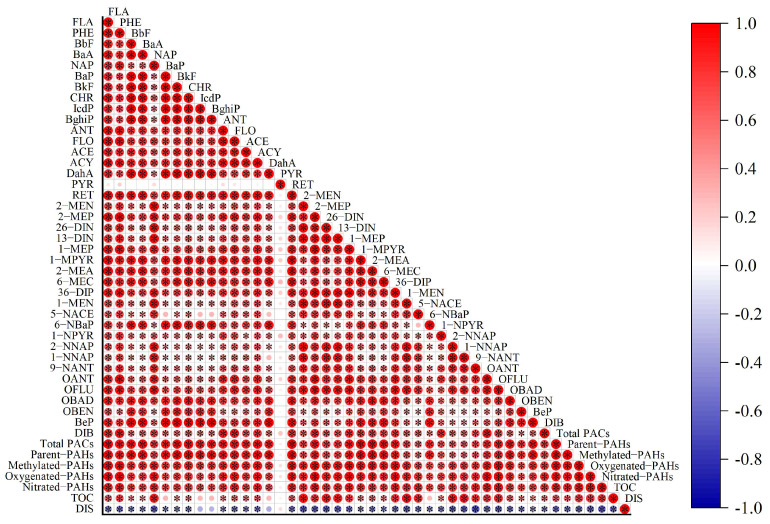
The correlation among PAC individuals, as well as their correlation with influencing factors (TOC and distance away from the coking plant). The size of the red circles is proportional to the degree of correlation. * indicates *p* < 0.05.

**Figure 6:**
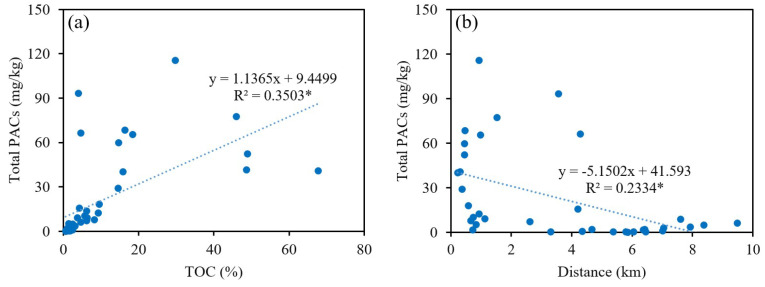
Total PACs’ concentration versus TOC (**a**) and distance away from the coking plant (**b**). * indicates a significant correlation, *p* < 0.05.

The spatial distribution of the concentration of total PACs and PAC groups was obtained using ArcGIS 10.1. It was observed that the concentration of methylated-PAHs, oxygenated-PAHs, and nitrated-PAHs featured a similar spatial distribution as total PACs did (Figure 4), namely, decreasing from the center of the coking plant outward. The similar spatial distribution could be further reflected by the significant positive correlation among PAC individuals (Figure 5), with the exception of PYR due to wood-burning sources outside the plant. Potential impacts of environmental pollutants are determined by not only the spatial distribution of those pollutants but also the population distribution. Figure 4f shows the spatial distribution of population-weighted PACs; in comparison with that of the total PACs in soil, it shared similar patterns, suggesting the critical role played by population factors in the process of health risk assessment and control of PACs in industrial parks. Thus, PAC pollution status over the residential area located southwest of the coking plant deserves attention, as a large number of people living therein are being affected more directly and heavily by emissions. Statistically negative correlations between concentration levels and distance away from the plant center were discerned (Figure 5, *p* < 0.05). The average concentration of total PACs within a 5 km radius of the plant (covering residential areas, villages, plant facilities, and arable land) was 34.9 mg/kg, sharply dropping to 2.7 mg/kg beyond 5 km (Figure 6b), indicating strong impacts of the coking emissions. Besides direct emissions, soil PAC contaminations are expected to be obviously affected by soil properties like soil organic matter and microbial communities [21,60,61].

PAC individuals, after being adsorbed by soil organic matter, undergo processes such as soil–air exchange, biodegradation, and migration. Therefore, TOC content is an important factor influencing their concentration and distribution [62]. The soil TOC content in this study ranged from 0.5% to 67.7%, with an average of 9.7%. As shown in Figure 6a, TOC content exhibited a significant positive correlation with total PACs (*p* < 0.05), and similar spatial distribution patterns were found between TOC and PAH groups. TOC was significantly positively correlated with 13 individuals of parent-PAHs and PAH derivatives, except for 6-NBaP. It is worth noting that the correlation between TOC and high-molecular-weight PACs, including BaP, IcdP, BghiP, DahA, and 6-NBaP, was not significant, which is not consistent with previous studies [24,63,64]. This discrepancy could be attributed to the source of samples and soil properties. The soil samples of previous studies, derived from roadside, farmland soil, and grassland, differ from those in this study. Moreover, this may be attributed to the differences in the sources of TOC and high molecular weight PACs, especially outside the plant. High molecular weight PACs are prone to formation during high-temperature combustion of gasoline and fossil fuels, whereas TOC mainly originates from natural sources and anthropogenic activities (including residential and agricultural emissions).

Wind condition, including wind direction and wind speed, is one of the key factors considered in the selection of sites for industrial parks to minimize their impact on air quality in downwind areas. Studies have indicated that wind patterns play a significant role in influencing air quality [65,66]. Here, based on meteorological data collected from a nearby weather station (36.18° N, 118.15° E) between 2018 and 2022, dominant wind directions, easterly and east-northeasterly, accounted for a total of 29.7% (16 wind-sector). This led to the deposition and accumulation of PAC individuals in areas adjacent to the plant, consistent with findings by Ren et al. [67] and Wu et al. [25]. Several studies have highlighted dense populations in the downwind area of industrial parks, calling for effective controls on health risks due to these industrial emissions [13,67,68,69]. The results here provide some insights for the design of new industrial parks.

### 3.4. Potential Emission Sources and PCA-MLR Analysis

The type and concentration of pollutants vary with emission sources. The PAC diagnostic ratios method has been widely applied to distinguish potential sources contributed [22,26]. Several typical ratios, including BaA/(BaA + CHR) and FLA/(FLA + PYR), are selected to look into potential sources of soil PACs [70,71,72,73,74,75], and ratios of 1-NPYR/PYR, 9-NANT/1-NPYR, OFLU/FLO, and OBAD/BaA are calculated to analyze potential source contributions of PAH derivatives. It is noted that due to the limitation of sample size, more robust models like Positive Matrix Factorization (PMF) are not run here.

**Figure 7:**
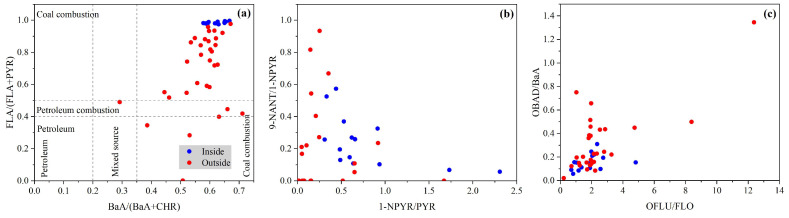
PAC diagnostic ratios and the corresponding potential emission sources inside and outside the coking plant. BaA/(BaA + CHR), FLA/(FLA + PYR), 1-NPYR/PYR, and 9-NANT/1-NPYR, indicated by (**a**,**b**), are used in distinguishing sources like coal combustion or petroleum. OFLU/FLO and OBAD/BaA, indicated by (**c**), are used in distinguishing primary emissions or secondary formation. The data ranges divided by dashed lines in (**a**) represent potential sources of PACs.

As seen from Figure 7, BaA/(BaA + CHR) and FLA/(FLA + PYR) ratios were at 0.59 and 0.78, respectively, both exceeding the thresholds of 0.35 and 0.50, indicating the source of coal combustion. It should be noted that the burning of straw in the years preceding the straw-burning ban also contributes significantly to soil PACs [76,77]. Both 1-NPYR/PYR and 9-NANT/1-NPYR ratios, 0.53 and 0.31, respectively, are attributed to vehicle emissions. From these results, the main sources of soil PACs in the study area are coal combustion and vehicle emissions. The values of OFLU/FLO and OBAD/BaA inside (1.90 and 0.16) and outside (2.49 and 0.31) the plant were higher than the corresponding observed values by direct emission in documents [73,78,79,80], indicating obvious contributions of secondary formation.

PCA is another common method for source apportionment, with previous studies typically categorizing sources in coking plants into combustion and vehicle emissions [22,24]. In this study, the PCA method has difficulties providing detailed source information because typical indicators for certain sources failed to reasonably distribute in components. However, PCA showed the ability to distinguish direct emissions and secondary formation [78]. The results of the present study show that the first principal component, accounting for 68.6% of the total variance, included 14 parent-PAHs and eight PAH derivatives. These 14 parent-PAHs were abundant, comprising 56.8% of the total PAC concentration, suggesting that the first component represented primary direct emissions. The second principal component, contributing 15.2% of the total variance, included four parent-PAHs (PHE, NAP, PYR, DIB) and 18 PAH derivatives (Table S3). Among the 18 PAH derivatives, nine were nitrated-PAHs and two were high-abundance oxygenated-PAHs (OANT, OFLU), which are typically products of secondary formation [30,73,81]. Hence, the second principal component represents secondary formation. PCA-MLR was employed here to further quantify their contributions. The results showed that the proportion of direct emission and secondary formation was 60.4% and 39.6%, respectively. The proportion of secondary formation accounted for in soil is scarcely reported over coking plants. However, in this study, secondary formation played a critical role in determining the PAC concentration in soil.

### 3.5. Exposure Risk Assessment

**Figure 8:**
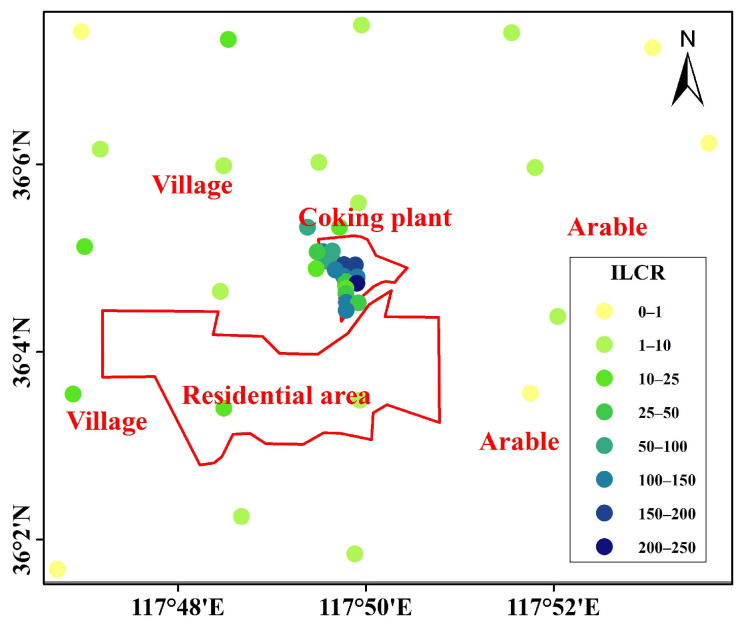
ILCR at sampling sites. Values are presented by uniformly multiplying 1.0 × 10^6^.

**Figure 9:**
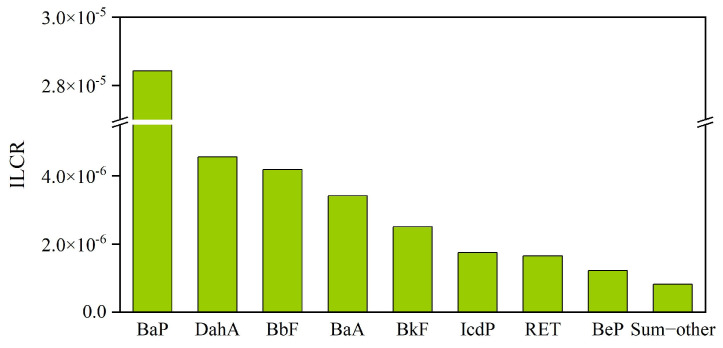
The contributions of PAC individuals to ILCR. Sum−other indicates individuals with ILCR less than 1.0 × 10^−6^, including methylated−PAHs, oxygenated−PAHs, and nitrated−PAHs.

Soil PACs can obviously affect human health via multiple pathways of dermal contact, ingestion, and inhalation of re-raised soil particles, with the former two being main intake pathways and respiratory exposure typically 2~5 orders of magnitude lower [64,82,83]. Soil PAC exposure risks have been widely studied in a number of previous studies, mostly on priority PAHs. Contributions of non-priority PAHs are less studied due to the short targeted compound list as well as the lack of reliable toxic data. Our results here suggested that dermal contact and ingestion are primary intake pathways, causing ILCRs of 3.8 × 10^−5^ and 1.0 × 10^−5^ attributed to the targeted priority and non-priority PAHs, respectively, and the respiratory exposure contribution was much lower (1.1 × 10^−7^), with proportions corresponding to 79.1%, 20.7%, and 0.2%, respectively (Table 2). The ILCR proportion contributed by priority PAHs and non-priority PAHs over the study was 93.6% (4.6 × 10^−5^) and 6.4% (3.1 × 10^−6^), respectively; thus, ILCR was dominated by priority PAHs. As shown in Figure 8, ILCR values of the majority sites outside the plant fell in the range of potentially low-risk level (1.0 × 10^−6^~1.0 × 10^−4^), while sampling sites located in the central plant exceeded the limitation of high-risk level (>1.0 × 10^−4^), which requires continuous attention. BaP, a highly carcinogenic individual, is mainly caused by the incomplete burning of fossil and biomass fuels. Due to its high concentration and high TEF in coking plant soils, BaP is one of the main PAC individuals contributing to the total ILCR (about 58.5% in the present study). Other species contributing largely to the ILCR were DahA, BbF, BaA, BkF, and IcdP (Figure 9).

The risk due to priority PAHs was expectedly higher than that due to non-priority ones; however, this does not mean the impact of non-priority PAHs is negligible, especially given the uncertainty in evaluating the exposure risk of these non-typical criteria compounds. There are so far only a few TEFs for non-priority PAHs [53,64], and the absence of TEFs for most non-priority PAHs would likely lead to an underestimation of exposure risk. Moreover, available TEFs for some non-priority PAHs vary largely in studies; for example, the TEF of BeP is employed to be 0.01 and 1.0, respectively, in two studies, causing significant discrepancies in further risk quantification [32,84]. It has been recognized that for some non-priority PAHs, their mutagenicity and carcinogenicity potentials are likely much higher than their corresponding parent ones [85], like some high molecular weight parent-PAHs (e.g., Dibenzo[a,e]fluoranthene, Dibenzo[a,i]pyrene, Dibenzo[a.l]pyrene) and nitrated derivatives. Yet, studies on exposure risk assessment of high-toxicity non-priority PAHs are limited and deserve more future work.

Previous studies have highlighted significant heterogeneity distributions of soil PACs across different regions [26,30,72]. Similarly, a strong heterogeneity of ILCR is observed in the coking plant, as compared to studies [23,26,45]. The ILCR reported in the literatures ranged from 2.9 × 10^−5^ to 4.1 × 10^−4^, with the highest and lowest values reported in studies in Beijing (North China) and Shaoguan (South China), respectively. ILCR values of all the coking plants exceeded the potentially low-risk level of 1.0 × 10^−6^, and some areas even exceeded the high-risk level of 1.0 × 10^−4^. We find most coking plants in previous studies are located in the outskirts or suburbs of cities with dense populations in their surroundings. Due to their long-term operations (more than 50 years) and high production capacity (1.0~2.0 million tons/year), PACs in soils are expected to accumulate. To mitigate exposure risks, effective measures should consider strengthening end-of-pipe treatment and enforcing stricter industry emission standards.

## 4. Conclusions

Emissions from coke production are a significant contributor to soil PAC contamination, with particular concern regarding surrounding residential areas due to the associated health risks. In this study, a typical coking plant, surrounded by residential areas, is focused on assessing the pollution status and health risks associated with parent-PAHs and their derivatives.

We concluded that the concentrations of soil parent-PAHs and their derivatives inside the coking plant were much higher than those outside. The individuals who determined the concentrations of PACs were found to be identical both inside and outside the plant, including Fluoranthene, Phenanthrene, Benzo(b)fluoranthene, Benzo(a)anthracene, and Naphthalene. Correlation analysis confirmed a strong positive relationship between individuals of parent-PAHs and their derivatives overall (*p* < 0.05), accompanied by similar spatial distribution patterns, indicating a common source of soil PACs. Based on the diagnostic ratio method, coal combustion and vehicle emissions were identified to be the main sources of soil PACs in the study area. Moreover, the PCA-MLR method quantitatively determined that direct emission and secondary formation accounted for 60.4% and 39.6% of PAC concentration, respectively, providing insights into the proportion of secondary formation of PACs in coking plant soil.

The residential area’s proximity to the coking plant carried a potential risk level of exposure. However, given that persistent production is yielding increasing exposure risk, continuous attention should be paid to the area. High concentrations of PACs were also identified in areas close to the coking plant and downwind locations, with these being the main contributing factors. This highlights the importance of carefully selecting sites for both industrial parks and residential areas.

## Figures and Tables

**Table 1:** Parameters employed for ILCR calculation.

Parameter	Unit	Child		Adolescent		Adult	
		Male	Female	Male	Female	Male	Female
Average body weight (BW)	kg	17.2	16.5	47.1	44.8	60.2	53.1
Exposure frequency (EF)	d/year	350	350	350	350	350	350
Exposure duration (ED)	year	6.0	6.0	14.0	14.0	30.0	30.0
Inhalation intake rate (IRair)	m^3^/d	10.9	10.9	17.7	17.7	17.5	17.5
Soil intake rate (IRsoil)	mg/d	200	200	100	100	100	100
Dermal surface exposure (SA)	cm^2^/d	1800	1800	5000	5000	5000	5000
Average life span (AT)	year	25,550	25,550	25,550	25,550	25,550	25,550
Soil dust produce factor (PEF)	m^3^/kg	6.2 × 10^9^	6.2 × 10^9^	6.2 × 10^9^	6.2 × 10^9^	6.2 × 10^9^	6.2 × 10^9^
Carcinogenic slope factor (CSF) Ingestion	(mg/kg·d)^−1^	7.3	7.3	7.3	7.3	7.3	7.3
Carcinogenic slope factor (CSF) Dermal	(mg/kg·d)^−1^	25	25	25	25	25	25
Carcinogenic slope factor (CSF) Inhalation	(mg/kg·d)^−1^	3.85	3.85	3.85	3.85	3.85	3.85
Particle-to-skin adherence factor (AF)	mg/cm^2^	0.2	0.2	0.2	0.2	0.2	0.2
Dermal adsorption fraction (ABS)	-	0.16	0.16	0.16	0.16	0.16	0.16

Note: ILCR less than 1.0 × 10^−6^ indicates no appreciable risk level; ILCR in the range 1.0 × 10^−6^~1.0 × 10^−4^ indicates potentially low-risk level; ILCR greater than 1.0 × 10^−4^ indicates high-risk level.

**Table 2:** The ILCR of the three pathways belongs to priority and non-priority PAHs.

Exposure Pathway	Priority PAHs	Non-Priority PAHs	Total
Dermal	3.6 × 10^−5^	2.5 × 10^−6^	3.8 × 10^−5^
Ingestion	9.4 × 10^−6^	6.4 × 10^−7^	1.0 × 10^−5^
Inhalation	1.1 × 10^−7^	7.2 × 10^−9^	1.1 × 10^−7^

## Data Availability

The raw data presented in this study are available upon request from the corresponding author.

## Supplementary Materials

The following supporting information can be downloaded at: https://www.mdpi.com/article/10.3390/toxics12030179/s1, Table S1: Instrument detection limits (IDLs), method recovery rates, and method detection limits (MDLs) of PAC individuals; Table S2: Concentration, detection rate, and proportion of PAC components in soil inside and outside the coking park; Table S3: Principal component analysis by employing priority PAHs and non-priority PAHs.
