# Supplementary material for: Pollution Characteristics and Health Risks of Polycyclic Aromatic Compounds (PACs) in Soils of a Coking Plant
Source: Toxics. 2024 Feb 27;12(3):179. doi: 10.3390/toxics12030179 (PMC10974274; doi:10.3390/toxics12030179)
Supplement: Supplementary file 1 [file toxics-12-00179-s001.zip › toxics-2849321-supplementary.pdf]

SUPPORTING INFORMATION FOR

Pollution Characteristics and Health Risk of Polycyclic Aromatic Compounds (PACs) in Soils of a Coking Plant

Yousong Zhou [1], Yuancheng Li [2], Donglei Fu [1, *], Yongqiang Zhang [1], Kai Xiao [1], Ke Jiang [1], Jinmu Luo [3], Guofeng Shen [1], Wenxin Liu [1], Shu Tao [1]

1. College of Urban and Environmental Sciences, Laboratory for Earth Surface Processes, Peking University, Beijing 100871, China
2. School of Public Health, Shandong First Medical University & Shandong Academy of Medical Science, Shandong Province 250117, China
3. Department of Earth and Atmospheric Sciences, Cornell University, Ithaca, New York 14853, United States

Yousong Zhou and Yuancheng Li contributed equally to this work
* Corresponding author: Dr. Donglei Fu, fdl2306386517@pku.edu.cn

Table S1. Instrument detection limits (IDLs), method recovery rates, and method detection limits (MDLs) of PAC individuals.

Abbreviation	Full name	IDLs (ng/mL)	Method recovery rates (%)	MDLs (pg/g)
NAP	naphthalene	0.039	102.3	1.0
2-MEN	2-methylnaphthalene	0.149	101.8	3.7
1-MEN	1-methylnaphthalene	0.072	98.4	1.8
26-DIN	2,6-dimethylnaphthalene	0.018	104.7	0.4
13-DIN	1,3-dimethylnaphthalene	0.012	132.7	0.2
ACY	acenaphthyleme	0.045	124.6	0.9
ACE	acenaphthene	0.024	113.4	0.5
FLO	fluorene	0.021	106.2	0.5
DIB	dibenzothiophene	0.008	130.6	0.2
PHE	phenanthrene	0.011	102.9	0.3
ANT	anthracene	0.041	113.7	0.9
2-MEP	2-methylphenanthrene	0.031	98.5	0.8
2-MEA	2-methylanthracene	0.073	78.8	2.3
1-MEP	1-methylphenanthrene	0.015	89.8	0.4
36-DIP	3,6-dimethylphenanthrene	0.087	99.6	2.2
FLA	fluoranthene	0.036	106.1	0.8
PYR	pyrene	0.155	87.6	4.4
RET	retene	0.079	89.3	2.2
1-MPYR	1-methylpyrene	0.03	67.7	1.1
BaA	benzo(a)anthracene	0.086	70.5	3.0
CHR	chrysene	0.131	81.1	4.0
6-MEC	6-methylchrysene	0.064	75	2.1
BbF	benzo(b)fluoranthene	0.061	76	2.0
BkF	benzo(k)fluoranthene	0.09	61.7	3.6
BeP	benzo(e)pyrene	0.146	79	4.6
BaP	benzo(a)pyrene	0.098	62.3	3.9
IcdP	indeno(l,2,3-cd)pyrene	0.078	66.1	3.0
DahA	dibenz(a,h)anthracene	0.182	68.5	6.6
BghiP	benzo(g,h,i)perylene	0.149	69.6	5.4
1-NNAP	1-nitronaphthalene	7.895	59.2	333.4
2-NNAP	2-nitronaphthalene	0.04	94.9	1.1
5-NACE	5-nitroacenaphthene	0.085	103.2	2.1
2-NFLU	2-nitrofluorene	0.216	72	7.5
9-NANT	9-nitroanthracene	0.077	61	3.2
9-NPHE	9-nitrophenanthrene	0.039	102.3	1.0
3-NPHE	3-nitrophenanthrene	0.149	101.8	3.7
3-NFLA	3-nitrofluoranthene	0.072	98.4	1.8
1-NPYR	1-nitropyrene	0.018	104.7	0.4
7-NBaA	7-nitrobenzo(a)anthracene	0.012	132.7	0.2
6-NBaP	6-nitrochrysene	0.045	124.6	0.9
OFLU	6-nitrobenzo(a)pyrene	0.024	113.4	0.5
OANT	9-fluorenone	0.021	106.2	0.5
OBEN	anthraquinone	0.008	130.6	0.2
OBAD	benzanthrone	0.011	102.9	0.3

Table S2. Concentration, detection rate, and proportion of PAC components in soil inside and outside the coking park

Component	Inside				Outside			
	Mean (μg/kg)	Range (μg/kg)	Detection rate (%)	Proportion (%)	Mean (μg/kg)	Range (μg/kg)	Detection rate (%)	Proportion (%)
FLA	5012.5	678-13213	100.0	10.99	638.6	0-9599	100.0	11.34
PHE	4332.7	657-10780	100.0	9.50	674.4	1-12727	100.0	11.97
BbF	3710.4	366-9890	100.0	8.14	278.8	14-1789	100.0	4.95
BaA	2943.2	375-9064	100.0	6.45	247.6	5-1913	100.0	4.40
NAP	2865.6	345-7127	100.0	6.28	351.4	4-5114	100.0	6.24
BaP	2430.7	234-7083	100.0	5.33	192.1	7-1124	100.0	3.41
BkF	2213.5	222-6109	100.0	4.85	153.1	6-1003	100.0	2.72
CHR	1735.8	220-4786	100.0	3.81	185.0	5-1307	100.0	3.28
IcdP	1485.3	134-4103	100.0	3.26	155.9	6-601	100.0	2.77
BghiP	1133.8	115-3172	100.0	2.49	187.1	6-871	100.0	3.32
ANT	864.7	129-2650	100.0	1.90	152.6	1-3054	100.0	2.71
FLO	649.0	201-1827	100.0	1.42	100.4	1-1659	100.0	1.78
ACE	405.0	129-1431	100.0	0.89	39.9	0-338	100.0	0.71
ACY	414.9	104-1057	100.0	0.91	46.9	0-707	100.0	0.83
DahA	380.1	36-1159	100.0	0.83	53.3	1-216	100.0	0.95
PYR	69.8	18-168	100.0	0.15	129.0	8-756	100.0	2.29
BeP	2671.6	288-7152	100.0	5.86	274.3	10-1531	100.0	4.87
DIB	167.4	25-552	100.0	0.37	43.2	0-997	100.0	0.77
RET	3474.9	495-9075	100.0	7.62	443.9	1-5400	100.0	7.88
2-MEN	1460.1	220-4346	100.0	3.20	177.6	1-1641	100.0	3.15
2-MEP	788.0	109-1873	100.0	1.73	135.5	1-1800	100.0	2.41
26-DIN	639.9	151-1780	100.0	1.40	101.2	2-919	100.0	1.80
13-DIN	507.2	120-1386	100.0	1.11	95.0	0-834	100.0	1.69
1-MEP	336.4	42-854	100.0	0.74	66.7	2-744	100.0	1.18
1-MPYR	204.9	34-514	100.0	0.45	46.1	1-394	100.0	0.82
2-MEA	198.5	25-632	100.0	0.44	33.5	1-275	100.0	0.60
6-MEC	158.5	28-468	100.0	0.35	44.4	1-264	100.0	0.79
36-DIP	129.2	17-352	100.0	0.28	28.6	0-234	100.0	0.51
1-MEN	57.4	12-174	100.0	0.13	7.3	0-66	100.0	0.13
OANT	1635.0	153-3923	100.0	3.58	290.7	7-5542	100.0	5.16
OFLU	1353.2	148-2835	100.0	2.97	137.2	10-1973	100.0	2.44
OBAD	455.2	34-1402	100.0	1.00	45.4	5-285	100.0	0.81
OBEN	354.8	16-1536	100.0	0.78	12.3	0-112	96.9	0.22
5-NACE	147.2	27-637	100.0	0.32	14.2	0-183	84.4	0.25
6-NBaP	99.2	12-287	100.0	0.22	5.6	0-75	18.8	0.10
1-NPYR	46.3	8-134	100.0	0.10	18.7	0-276	59.4	0.33
2-NNAP	19.4	7-48	100.0	0.04	8.5	4-26	100.0	0.15
1-NNAP	15.7	8-35	100.0	0.03	8.8	5-19	100.0	0.16
7-NBaA	9.5	0-43	46.7	0.02	0.6	0-12	6.3	0.01
2-NFLU	8.9	4-20	100.0	0.02	1.4	0-8	25.0	0.03
9-NANT	8.2	4-14	100.0	0.02	2.3	0-15	37.5	0.04
3-NFLA	5.7	0-16	53.3	0.01	0.8	0-11	9.4	0.01
9-NPHE	4.7	0-8	86.7	0.01	1.2	0-8	21.9	0.02
3-NPHE	3.2	0-6	66.7	0.01	1.0	0-7	18.8	0.02

Table S3. Principal component analysis by employing priority PAHs and non-priority PAHs

Component	PC1	PC2
FLA	0.783	0.598
PHE	0.577	0.762
BbF	0.955	0.245
BaA	0.954	0.263
NAP	0.318	0.914
BaP	0.975	0.149
BkF	0.962	0.218
CHR	0.937	0.325
IcdP	0.975	0.155
BghiP	0.97	0.17
ANT	0.733	0.486
FLO	0.688	0.61
ACE	0.742	0.453
ACY	0.748	0.556
DahA	0.976	0.149
PYR	-0.018	0.206
RET	0.851	0.505
2-MEN	0.25	0.902
2-MEP	0.568	0.795
26-DIN	0.319	0.901
13-DIN	0.34	0.9
1-MEP	0.578	0.783
1-MPYR	0.863	0.445
2-MEA	0.819	0.51
6-MEC	0.86	0.395
36-DIP	0.536	0.793
1-MEN	0.257	0.903
5-NACE	0.162	0.825
6-NBaP	0.918	0.144
1-NPYR	0.406	0.557
2-NNAP	0.151	0.935
1-NNAP	0.124	0.931
7-NBaA	0.735	0.006
2-NFLU	0.289	0.845
9-NANT	0.244	0.862
3-NFLA	0.216	0.74
9-NPHE	0.235	0.799
3-NPHE	0.044	0.804
OANT	0.432	0.822
OFLU	0.521	0.825
OBAD	0.79	0.463
OBEN	0.771	0.203
BeP	0.958	0.247
DIB	0.466	0.662
